# Exploring neonatal care implications in low Apgar scores and therapeutic hypothermia

**DOI:** 10.1590/1806-9282.20250160

**Published:** 2025-07-07

**Authors:** Yijia Zhang, Qing He

**Affiliations:** 1 Fuyang District Maternal and Child Health Hospital, Department of Neonatology – Hangzhou, China.

Dear Editor,

I read with great interest the study by Carlstron et al.^1^, which investigates the relationship between maternal and perinatal complications and the use of therapeutic hypothermia in newborns with low Apgar scores at the 5th min. The study sheds light on important neonatal care practices, but it also opens avenues for further discussion on optimizing outcomes for neonates requiring specialized care.

The findings highlight that therapeutic hypothermia is not significantly associated with maternal or perinatal complications, yet it is vital to consider the broader implications of this treatment for neonatal outcomes. The study reports longer neonatal intensive care unit (NICU) stays and a higher prevalence of intracranial hemorrhage and vasoactive drug use in newborns undergoing therapeutic hypothermia. These outcomes emphasize the necessity of close monitoring and tailored interventions in neonates with low Apgar scores to minimize risks and improve recovery trajectories.

Furthermore, the study underscores the importance of identifying risk factors for low Apgar scores, such as prematurity and perinatal asphyxia, to refine neonatal care protocols. Research has consistently shown that early identification and intervention for neonates at risk can not only improve short-term outcomes but also reduce the burden of long-term complications, such as neurodevelopmental delays. For instance, a retrospective cohort study found that preterm infants with low Apgar scores are significantly more likely to require extensive resuscitation efforts and face higher mortality rates compared to their full-term counterparts^2^. This is particularly critical in facilities catering to low-risk pregnancies, where the occurrence of low Apgar scores might be less frequent but carries substantial implications when present. In addition, therapeutic hypothermia has been demonstrated to reduce the severity of hypoxic-ischemic encephalopathy (HIE), as highlighted in the discussion. Studies indicate that therapeutic hypothermia initiated within 6 h after birth can lead to improved neurodevelopmental outcomes in affected infants^3,4^. However, its accessibility and application in resource-limited settings remain a challenge. Expanding access to this life-saving intervention through cost-effective cooling devices and standardized training for healthcare providers could bridge the gap in care disparities. For example, implementing a systematic approach known as "HIE Code" in these settings could optimize care practices and improve outcomes for infants suffering from HIE^4^. Finally, it is noteworthy that the study advocates for the use of Apgar scores at the 5th min as a criterion for initiating therapeutic hypothermia. Recent findings suggest that a low Apgar score at this time is more relevant for identifying infants eligible for therapeutic hypothermia than other criteria, such as metabolic acidosis^5^. Future research could explore integrating additional biomarkers or imaging techniques to enhance the specificity and sensitivity of identifying neonates who would most benefit from this intervention. For instance, combining Apgar scores with serum lactate levels or brain imaging could provide a more comprehensive assessment of an infant's condition and guide timely therapeutic decisions^3,6^. This integrated approach not only aims to improve immediate neonatal care but also seeks to mitigate long-term neurological deficits associated with perinatal asphyxia.

In conclusion, the study by Carlstron et al. is a valuable contribution to neonatal care literature, offering insights into managing neonates with low Apgar scores and advancing the use of therapeutic hypothermia. Further exploration of its long-term implications and adaptation in diverse clinical settings will be crucial for improving neonatal health outcomes globally.
